# Ethyl Acetate Fraction from *Leandra dasytricha* (A. Gray) Cong. Leaves Promotes Vasodilatation and Reduces Blood Pressure in Normotensive and Hypertensive Rats

**DOI:** 10.1155/2021/7203934

**Published:** 2021-11-15

**Authors:** Rita de Cassia Vilhena da Silva, Luísa Nathália Bolda Mariano, Eleine Renata Bidinha, Camila Leandra Bueno de Almeida, Valdir Cechinel-Filho, Vanessa Samudio Santos Zanuncio, Denise Brentan Silva, Arquimedes Gasparotto Junior, Priscila de Souza

**Affiliations:** ^1^Postgraduate Program in Pharmaceutical Sciences, Nucleus of Chemical-Pharmaceutical Investigations, University of Vale do Itajaí, Itajaí, Brazil; ^2^Laboratory of Natural Products and Mass Spectrometry, Faculty of Pharmaceutical Sciences, Food and Nutrition, Federal University of Mato Grosso do Sul, Campo Grande, MatoGrosso do Sul, Brazil; ^3^Laboratory of Cardiovascular Pharmacology, Faculty of Health Sciences, Federal University of Grande Dourados, Dourados, MatoGrosso do Sul, Brazil

## Abstract

*Leandra dasytricha* (A. Gray) Cong. is widely distributed in the south of Brazil and is commonly used for cardiovascular and kidney ailments. For this study, we used male Wistar normotensive rats (NTRs) and spontaneously hypertensive rats (SHRs) to verify the effects of the ethyl acetate fraction (EAF) obtained from *L. dasytricha* leaves on isolated aorta relaxation and in the arterial blood pressure. The EAF was analyzed by LC-DAD-MS, and several components were annotated, including hydrolysable tannins, triterpenes, and *O*- and *C*-glycosylated dihydrochalcones, such as the most intense ion peak relative to *C*-hexosyl phloretin (nothofagin; compound number **13**). The EAF caused a concentration and endothelium-dependent relaxation of the aorta in both NTRs and SHRs. This effect was abolished in the endothelium-denuded aorta. L-NAME, a nonselective nitric oxide synthase inhibitor, and ODQ, a soluble guanylate cyclase inhibitor, entirely blocked the EAF-induced relaxation. The presence of a muscarinic receptor antagonist or a cyclooxygenase inhibitor did not alter the EAF's effectiveness in relaxing the aorta. The preincubation with tetraethylammonium, a Ca^2+^-activated K^+^ channel blocker, and with 4-aminopyridine, a voltage-dependent K^+^ channel blocker, significantly interfered with the EAF's relaxation. However, the incubation with glibenclamide, an ATP-sensitive K^+^ channel blocker, and barium chloride, an inward-rectifier K^+^ channel blocker, did not interfere with the EAF-induced relaxation. The EAF treatment also caused a dose-dependent decrease in the mean arterial pressure, systolic arterial pressure, and diastolic arterial pressure of both NTRs and SHRs, without significantly interfering with heart rate values. In conclusion, this study demonstrated the EAF-induced vasorelaxant and hypotensive actions, primarily dependent on the endothelium function and mainly with the participation of the nitric oxide and Ca^2+^-activated and voltage-dependent K^+^ channels.

## 1. Introduction

According to data from the American Heart Association (AHA), heart diseases are the leading causes of death globally. In 2019, nearly 18.6 million people worldwide died from cardiovascular diseases (CVDs) [1], hypertension being the most common risk factor [2]. The pathogenesis of hypertension involves many factors, and the treatment requires a healthy lifestyle and multiple therapies in a large majority of patients. The main objective in the treatment of hypertension is to prevent the occurrence of damage to the blood vessels and heart caused by sustained high blood pressure levels. A slight reduction in systolic blood pressure is enough to decrease mortality levels and cardiovascular risks [3]. There are several classes of antihypertensive drugs; however, the failure to effectively control blood pressure can occur for several reasons, including suboptimal treatment and disease progression, with nonadherence to the treatment being responsible for 50 to 80% of unsatisfactory pressure reduction [4–6]. For these reasons, cardiovascular disease risks remain increased in hypertensive patients. Alternative approaches, such as herbal preparations with higher efficacy and lower toxicity, are sought to improve treatments and are also used to prevent cardiovascular complications [7].

*Leandra dasytricha* (A. Gray) Cong., popularly known as “pixirica,” belongs to the Melastomataceae family, which is widely distributed in the south of Brazil [8]. In addition to *L. dasytricha,* other species of *Leandra* have also shown pharmacological effects*. L. lacunosa* had a hypoglycemic effect in alloxan-induced diabetes in rats, and *L. chaetodon* revealed an antifungal activity [9, 10]. Recently, the ethyl acetate fraction isolated from *L. dasytricha* leaves exhibited the ability to increase the urine volume when orally given to rats. Its isolated compound nothofagin presented diuretic, natriuretic, and renal protective effects in both normotensive and hypertensive rats [11, 12]. In addition, nothofagin induced endothelium-dependent vasodilation in renal arteries [13].

Although *L. dasytricha* is commonly used for cardiovascular and kidney ailments [11, 12], studies with its preparations are still scarce. Based on the premise of developing new therapeutic alternatives for the treatment of cardiovascular diseases, this study extends the investigations from a semipurified fraction obtained from the leaves of *L. dasytricha* and demonstrates the chemical profile of this preparation. In this context, it became interesting to evaluate the possible vasodilator and hypotensive properties in both normotensive and hypertensive rats. Spontaneously hypertensive rats (SHRs) are developed by selective breeding of Wistar Kyoto rats for high blood pressure to develop hypertension [14]; they are considered a suitable animal model of essential or primary human hypertension and have been widely used to study cardiovascular diseases [15].

## 2. Materials and Methods

### 2.1. Drugs and Chemicals

Atropine, acetylcholine chloride (ACh), indomethacin, N*ω*-nitro-l-arginine methyl ester (L-NAME), H-[1,2,3]oxadiazolo[4,3-alpha]quinoxalin (ODQ), phenylephrine hydrochloride (PE), tetraethylammonium (TEA), glibenclamide (GLI), 4-aminopyridine (4-AP), barium chloride (BaCl_2_), and all salts used to prepare the Krebs solution were purchased from Merck & Co. (Kenilworth, New Jersey, USA).

### 2.2. Phytochemical Procedures

The leaves of *Leandra dasytricha* (A. Gray) Cong. were collected from Camboriú, SC, Brazil (May 2014). Prof. Dr. Oscar Iza (UNIVALI) identified the plant species, and a voucher specimen has been deposited in the Barbosa Rodrigues Herbarium under number VCF 145. The preparation of the extract and the fractions were described by de Almeida et al. [11]. Briefly, fresh leaves of *L. dasytricha* were macerated at room temperature in methanol for 7 days. The ethyl acetate fraction (EAF) was obtained from the partition of the crude methanolic extract of *L. dasytricha* leaves by adding the ethyl acetate solvent.

### 2.3. LC-DAD-MS Analysis of *L. dasytricha*

The ethyl acetate fraction (EAF) of *L. dasytricha* leaves was solubilized in methanol and water (7 : 3, v/v) at a concentration of 2 mg/mL. Subsequently, this solution was filtered through a syringe filter (0.22 *μ*M pore, membrane PTFE, Millex^®^ filters) and analyzed by liquid chromatography coupled to a diode array detector and mass spectrometer (LC-DAD-MS) using a Kinetex C-18 column (2.6 *μ*m, 150 × 2.2 mm, Phenomenex) with a precolumn of the same stationary phase material. The mass spectrometer MicrOTOF-Q III (Bruker Daltonics) was provided with an electrospray ionization source and quadrupole-time-of-flight analyzers. The elution gradient profile was the same reported by Tolouei et al. [16]. The mobile phase was composed by acetonitrile and ultrapure water with 0.1% formic acid. The oven temperature was 50°C, and the volume of injection was 2 *μ*L.

The analyses were obtained by positive and negative ion modes (*m/z* 120–1200). Additionally, the compounds were monitored at wavelengths ranging from 240 to 800 nm. The annotation of the constituents from EAF was based on MS data (accurate mass and the ion fragmentation pathway) and UV data compared to data reported in the literature. The molecular formula of each compound was determined based on the mass errors within ±5 ppm and mSigma below 30. The authentic standards (gallic acid, catechin, and ellagic acid) were injected to confirm the identification of some metabolites.

### 2.4. Animals

Male normotensive Wistar rats (NTRs) and male spontaneously hypertensive rats (SHRs), twelve weeks old (250–300 g), provided by Universidade do Vale do Itajaí (UNIVALI), were kept at a constant temperature (22 ± °C) under a 12 h light/12 h dark cycle. The animal had free access to water and feed. The experiments were approved by the Ethical Committee for the Care and Use of animals of UNIVALI (authorization *n*°. 045/16 and 014/21), which followed the guidelines of the Brazilian National Council for Control of Animal Experimentation.

### 2.5. Isolation, Preparation of Rat Aorta, and Vascular Reactivity Studies

The thoracic aorta was isolated from male NTR and male SHR as previously described by Da Silva et al. [17].Briefly, the thoracic aorta was removed under anesthesia, cleaned of fat and connective tissue, and cut into rings (3-4 mm length). They were kept in organ baths containing Krebs solution (composition in mM : NaCl 115.3, KCl 4.9, CaCl_2_·2H_2_O 1.46, KH_2_PO_4_ 1.2, MgSO_4_ 1.2, d-glucose 11.1, and NaHCO_3_ 25), pH 7.4, maintained at 37°C under a resting tension of 1 g, and continuously aerated with 95% O_2_ and 5% CO_2_. Tension changes were obtained through isometric transducers coupled to DATAQ Instruments data acquisition hardware connected to a computer with specific software integration (WinDaq software, DATAQ Instruments, Akron, Ohio, USA). The rings remained at an equilibrium period of 60 min, with solution changes every 15 min. Then, the preparations were contracted with a potassium chloride solution (KCl: 60 mM) to test their responsiveness. To confirm the presence of a functional endothelium, a further 30 min stabilization period was expected. A new contraction was induced by adding phenylephrine (PE: 1 *μ*M), followed by administering 1 *μ*M acetylcholine (ACh). Only rings that showed relaxation equal to or greater than 80% were considered with functional endothelium. For preparations devoid of endothelium, this was mechanically removed by gently scraping the endothelial cells away from the intima. A new 30 min interval was expected for stabilization, and the aortic rings were then exposed to cumulative concentrations of EAF (10 to 500 *μ*g/mL) in preparations with and without functional endothelium at the plateau of the PE-induced contraction. To study the mechanisms involved in the vasorelaxant effect induced by the EAF, the rings were preincubated (30 min) before the addition of PE, with the following drugs: L-NAME (100 *μ*M, a nonselective NO synthase inhibitor), ODQ (100 *μ*M, an inhibitor of soluble guanylyl cyclase), atropine (1 *μ*M, a muscarinic receptor antagonist), indomethacin (1 µM, a cyclooxygenase inhibitor), and with the K^+^ channel blockers, which are as follows: tetraethylammonium (TEA, 1 mM, a calcium-activated K^+^ channel blocker) or TEA (10 mM, a nonselective K^+^ channel blocker), glibenclamide (10 *μ*M, an ATP-sensitive K^+^ channel blocker), 4-aminopyridine (4-AP, 1 mM, a voltage-sensitive K^+^ channel blocker), or barium chloride (BaCl_2_, 10 *μ*M, a nonselective inward-rectifier K^+^ channel blocker). The cumulative concentration curve of EAF was obtained in the presence of these drugs or just the vehicle.

### 2.6. Blood Pressure Analysis

The blood pressure study was conducted, as previously described by Da Silva et al. [18]. After anesthesia with ketamine/xylazine (80/10 mg/kg; by intramuscular route), the left femoral vein was cannulated with a polyethylene catheter filled with physiological saline solution (0.9%) for administration of heparin to prevent clot formation. The left carotid artery was isolated from the vagus nerve for insertion of a polyethylene catheter, which was connected to a pressure transducer coupled to an AECAD 04P recording system, running the software AQCAD 2.3.7 (Bonther, Ribeirão Preto, Brazil). Blood pressure was stabilized for 15 min with continuous real-time blood pressure recording. After that, three doses of EAF (0.3, 1, and 3 mg/kg), acetylcholine (10 nmol/kg), or vehicle were administered to the rats. All doses were given in a 200 µL bolus through duodenal access. Changes in mean arterial pressure (MAP), systolic arterial pressure (SAP), diastolic arterial pressure (DAP), and heart rate (HR) were recorded and compared between groups.

### 2.7. Statistical Analysis

All data are expressed as the mean ± standard error of the mean (SEM) from six preparations or animals per group. One- or two-way analysis of variance (ANOVA) followed by Bonferroni's multiple comparison test was performed using GraphPad Prism version 8 for Windows (GraphPad Software, La Jolla, CA, USA). A *p*value less than 0.05 was considered statistically significant.

## 3. Results

### 3.1. Chemical Analyses by LC-DAD-MS

**Figure 1:**
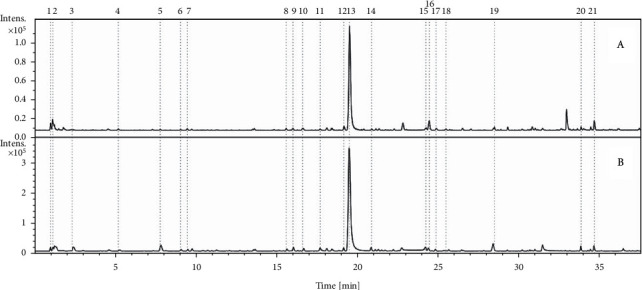
Base peak chromatograms obtained in positive (a) and negative ion mode (b) chromatograms from EAF.

The EAF was analyzed by LC-DAD-MS, and twenty-one compounds were annotated (Table 1 and Figure 1). The compounds were annotated based on their spectral data (MS, MS/MS, and UV) compared with data reported in the literature, and standards were used to confirm some compounds when possible.

The peaks **1** and **2** were putatively annotated as di-*O*-hexoside and shikimic acid, while the compounds **3**, **5,** and **6** presented similar UV spectra, and from the accurate mass, their molecular formulas were determined as C_7_H_6_O_5_, C_8_H_8_O_5_, and C_15_H_14_O_6_. In addition, **5** also revealed the product ion at *m/z* 168 [M-H-^•^CH_3_]^-• 19^, suggesting a methoxyl group, and **3** and **6** were confirmed by the authentic standard. Thus, **3**, **5**, and **6** were annotated as gallic acid, methyl gallate, and catechin.

The peaks **4**, **7**, and **8** showed similar UV (*λ*_max_ ≈ 275 nm), and they revealed a molecular formula with high number of carbons such as C_41_H_26_O_26_. They revealed fragment ions at *m/z* 301 and 169 which are relative to ellagic acid, confirming the hexahydroxydiphenoyl (HHDP) and gallic acid (Singh et al., 2016). For example, the successive losses of galloyl (152 *u*) from *m/z* 635 [M-H]^−^ (**8**) yielded the fragment ions *m/z* 465 [M-H-152-H_2_O]^−^ and 313 [M-H-152-2xH_2_O]^−^. The compounds **4**, **7,** and **8** were annotated as the hydrolysable tannins castalagin isomer, acutissimin A or B, and tri-*O*-galloyl hexoside, respectively.

The peaks **11** and **16** revealed the absorption bands in the UV spectra at the wavelength ≈ 280 and 368 nm, which is compatible with the chromophore of ellagic acid. The compound **11** (*m/z* 300.9996 [M-H]^−^, C_14_H_6_O_8_) was confirmed by ellagic acid as the authentic standard. The protonated ion from **16** revealed losses of 15 *u* (*m/z* 330 [M + H-^•^CH_3_]^+•^ and 315 [M + H-2x^•^CH_3_]^+•^), suggesting the methoxyl substituents [19], so it was annotated as tri-O-methyl ellagic acid.

The compounds **9-10**, **12-13**, **15**, and **17-19** revealed spectral data compatible to dihydrochalcones, such as the UV data. The characteristics of losses of 180 (hexosyl + H_2_O molecules) and 152 (galloyl), 42 *u* (cetene) and subsequent losses of 90 and 120 *u* suggest the substituents *O*-hexosyl, galloyl, acetyl, and *C*-hexosyl [20–23]. They were annotated as *O*-hexosyl phloretin (**9**), *C*-hexosyl hydroxyphlorizin (**10**), di-*C*-hexosyl phloretin (**12**), *C*-hexosyl phloretin (nothofagin) (**13**), *C*-hexosyl phloretin (**15**), *O*-galloyl *C*-hexosyl phloretin (**17**), *O*-acetyl *C*-hexosyl phloretin (**18**), and phloretin (**19**). The molecular structure of the major peak identified in the ethyl acetate fraction from *L. dasytricha *, nothofagin (13), can be seen in Figure S1 (supplementary material).

### 3.2. Effect of EAF from *L. dasytricha* in Thoracic Aorta Rings Precontracted with Phenylephrine

**Figure 2:**
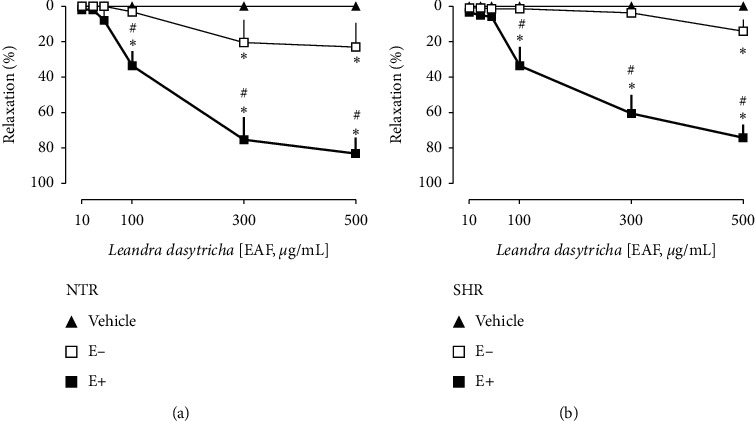
Vasorelaxation induced by EAF in the aorta of Wistar normotensive rats (NTRs) and spontaneously hypertensive rats (SHRs). Concentration-response curves were determined in endothelium-intact (E+) or endothelium-denuded (E-) rings. (a) The EAF-induced vasorelaxation in the NTR aorta. (b) EAF-induced vasorelaxation in the SHR aorta. Statistical analyses were performed using a two-way analysis of variance followed by Bonferroni's multiple comparison test. ^*∗*^*p* < 0.05 when compared to the vehicle (closed triangles), and ^#^*p* < 0.05 when compared to E- (opened squares).

The cumulative addition of EAF caused a concentration-dependent vasorelaxant effect in the endothelium intact aortic rings of both NTRs and SHRs. The maximal relaxation (*R*_max_) values in endothelium intact preparations were 83.22 ± 9.05% in NTRs and 74.87 ± 7.30% in SHRs (Figures 2(a) and 2(b), respectively). The vasodilator effect in the endothelium-denuded aorta was only 27.07 ± 16.54% in NTRs and 16.80 ± 8.09% in SHRs, indicating that the endothelium is essential for the relaxation induced by EAF. The vehicle (Krebs solution), represented in all images by the closed rectangle symbol, did not affect the aortic rings tonus.

### 3.3. Involvement of Nitric Oxide in the Vasorelaxant Activity of EAF

**Figure 3:**
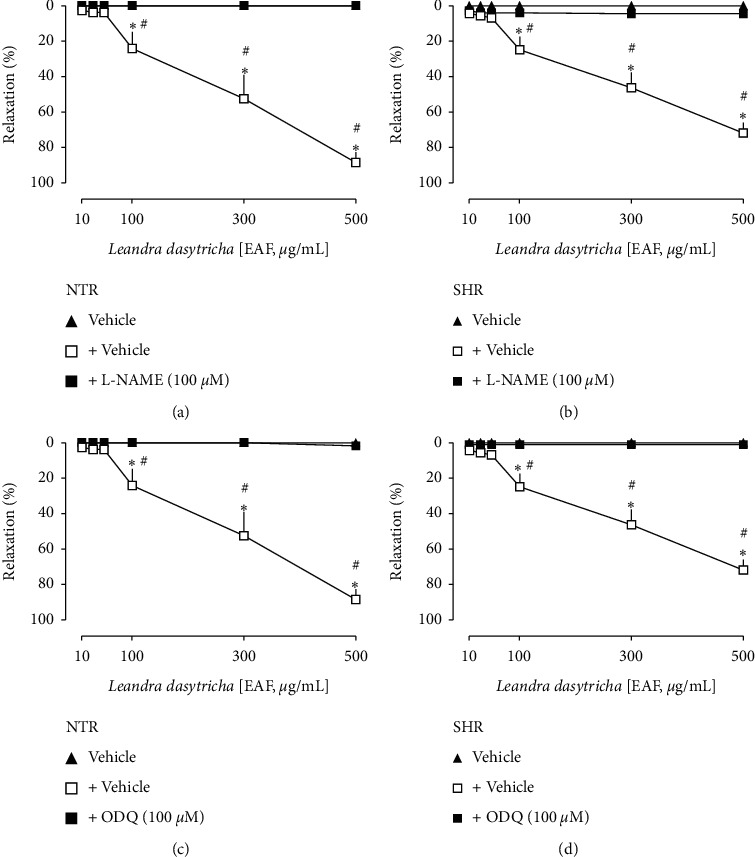
Vasorelaxation induced by EAF in the aorta of Wistar normotensive rats (NTRs) and spontaneously hypertensive rats (SHRs) in the absence or presence of L-NAME (a, b) and ODQ (c, d). Statistical analyses were performed using a two-way analysis of variance followed by Bonferroni's multiple comparison test. ^*∗*^*p* < 0.05 when compared to the vehicle (closed triangles), and ^#^*p* < 0.05 when compared to the vehicle-only incubation (opened squares).

**Figure 4:**
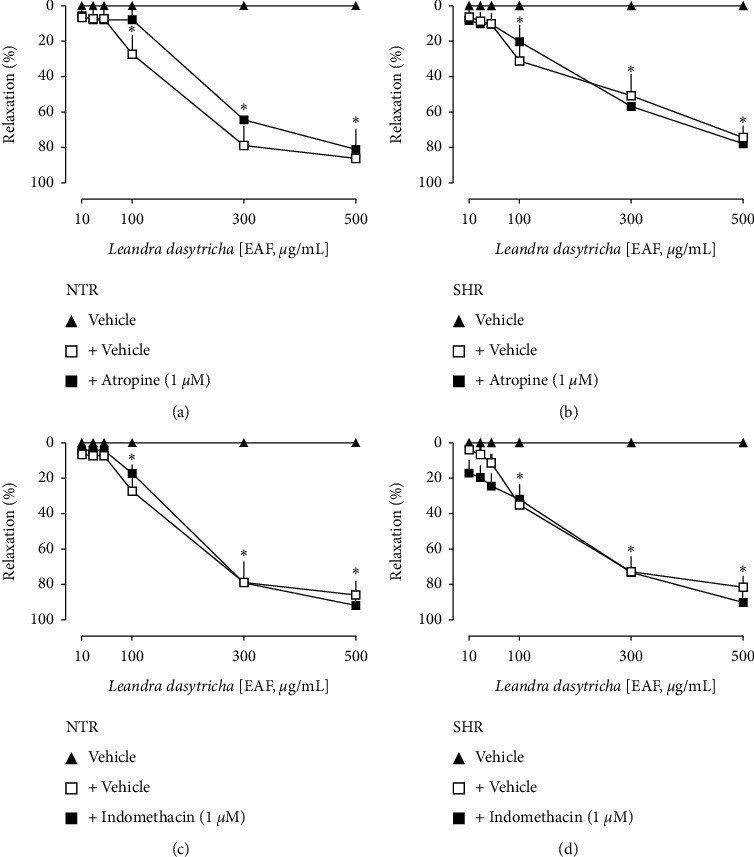
Vasorelaxation induced by EAF in the aorta of Wistar normotensive rats (NTRs) and spontaneously hypertensive rats (SHRs) in the absence or presence of atropine (a, b) and indomethacin (c, d). Statistical analyses were performed using a two-way analysis of variance followed by Bonferroni's multiple comparison test. ^*∗*^*p* < 0.05 when compared to the vehicle (closed triangles).

The nonselective nitric oxide synthase inhibitor L-NAME, as well as the soluble guanylate cyclase inhibitor ODQ, entirely blocked the EAF-induced relaxation in both NTR and SHR aorta rings (Figure 3). Besides, it can be seen in Figure 4 that neither atropine nor indomethacin modified the EAF-induced vasodilation response in the aorta of NTRs and SHRs.

### 3.4. Effect of K^+^ Channel Blockers on EAF-Induced Relaxation

**Figure 5:**
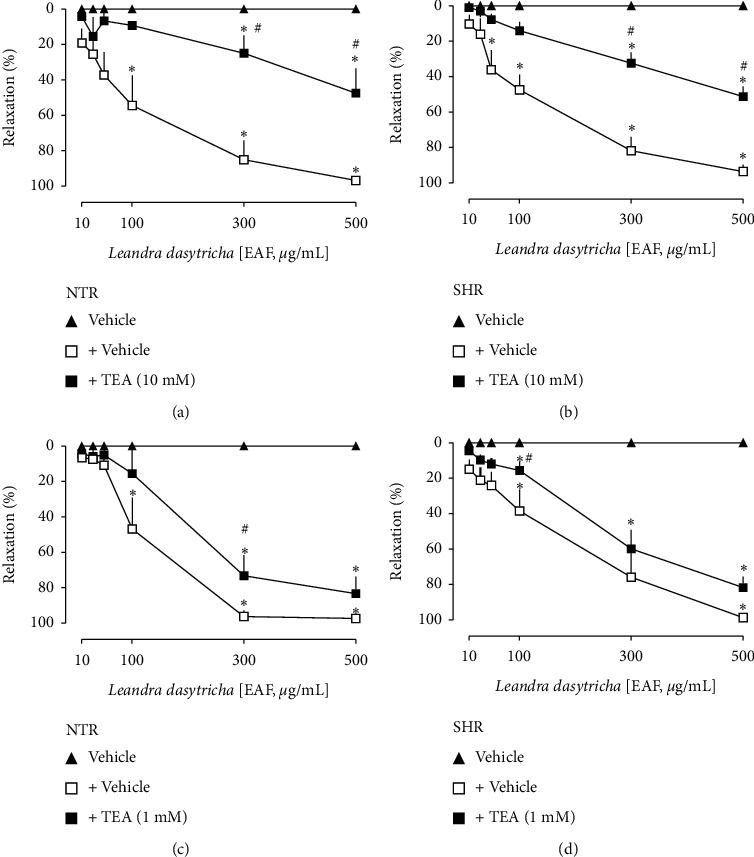
Vasorelaxation induced by EAF in the aorta of Wistar normotensive rats (NTRs) and spontaneously hypertensive rats (SHRs) in the absence or presence of TEA 10 mM (a, b) and TEA 1 mM (c, d). Statistical analyses were performed using a two-way analysis of variance followed by Bonferroni's multiple comparison test. ^*∗*^*p* < 0.05 when compared to the vehicle (closed triangles), and ^#^*p* < 0.05 when compared to the vehicle-only incubation (opened squares).

**Figure 6:**
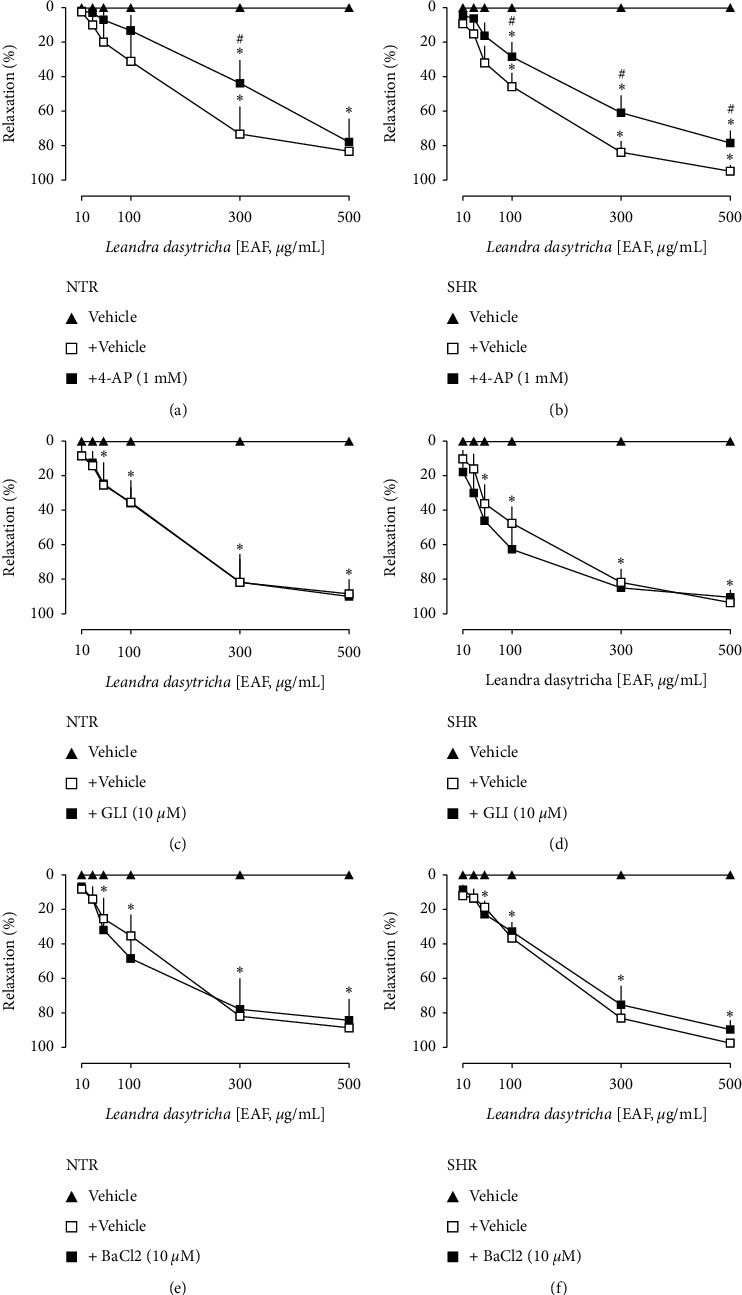
Vasorelaxation induced by EAF in the aorta of Wistar normotensive rats (NTRs) and spontaneously hypertensive rats (SHRs) in the absence or presence of 4-AP (a, b), glibenclamide (c, d), and BaCl_2_ (e, and f). Statistical analyses were performed using a two-way analysis of variance followed by Bonferroni's multiple comparison test. ^*∗*^*p* < 0.05 when compared to the vehicle (closed triangles), and ^#^*p* < 0.05 when compared to the vehicle-only incubation (opened squares).

The EAF-induced relaxation was significantly reduced in the presence of the nonselective potassium channel blocker (TEA, 10 mM) in both NTR and SHR preparations (Figures 5(a) and 5(b), respectively). Besides, TEA at a concentration of 1 mM (Figures 5(c) and 5(d)), which acts as a Ca^2+^-activated K^+^ channel blocker, reduced the relaxation response of EAF in both NTR and SHR aortas. The preincubation with 4-aminopyridine (4-AP), a voltage-dependent K^+^ channel blocker, significantly interfered with the EAF's relaxation in both NTR and SHR preparations (Figures 6(a) and 6(b), respectively). However, the incubation with glibenclamide (10 *μ*M), an ATP-sensitive K^+^ channel blocker, and barium chloride (10 *μ*M), an inward-rectifier K^+^ channel blocker, did not interfere with the EAF-induced relaxation (Figures 6(c)–6(f)).

### 3.5. EAF Reduces Mean and Systolic Blood Pressure in Both Normotensive and Hypertensive Rats

As depicted in Table 2, the EAF treatment at doses of 1 and 3 mg/kg, but not 0.3 mg/kg, triggered a dose-dependent decrease in the blood pressure of NTRs, with a reduction of∼24 mm Hg (at the highest dose tested) in the values of MAP, SAP, and DAP. Regarding the EAF treatment in SHR, a dose-dependent decrease in the blood pressure was also observed, with a reduction of ∼21 mm Hg in the values of MAP, SAP, and DAP at the highest dose tested. The values obtained with the administration of acetylcholine (10 nmol/kg) are also shown in Table 2 as the positive control of the experiment. None of the treatments significantly interfered with heart rate values (data not shown).

## 4. Discussion

In the present study, we showed that a semipurified fraction obtained from the leaves of *Leandra dasytricha* (A. Gray) Cong. induced a concentration-dependent relaxation in the aorta preparations, as well as dose-dependent hypotensive and antihypertensive effects in NTRs and SHRs, respectively. Due to the similarity with human arterial hypertension pathogenesis, SHRs have become the animals of choice for screening antihypertensive agents [15, 24]; thus, our study evidences a meaningful fraction-mediated action in an established disease model with translation into clinical practice.

The analyses by LC-DAD-MS from EAF revealed several compounds that had not been reported previously in *L. dasytricha*; thus, we here expanded the chemical knowledge of this medicinal species. Between the compounds annotated, we highlighted hydrolysable tannins, triterpenes, and mainly dihydrochalcones, such as the most intense ion peak C-hexosyl phloretin (nothofagin). This dihydrochalcone has been reported as a diuretic and natriuretic with potassium-sparing effects [11, 12].

The EAF-induced vasorelaxation was blunted with the endothelium removal, indicating that the integrity of the endothelium is necessary to EAF's vasodilation. To investigate the mechanism of endothelium-dependent relaxation, the vessels were pretreated with L-NAME, which completely blocked the vasorelaxation response, indicating NO's participation in the vasodilator response produced by EAF. Endothelial cells may cause vasorelaxation by releasing vasoactive substances called endothelium-derived relaxing factor (EDRF), identified as NO [25]. NO production and release is mediated through the upregulation of the endothelium nitric oxide synthase (eNOS) activity. NO diffuses into the vascular smooth muscle cells where it stimulates the soluble guanylate cyclase (sGC) that increases cyclic guanosine monophosphate (cGMP) concentration, which in turn activates a GMP-dependent protein kinase (PKG) that can phosphorylate and inhibit the myosin light chain kinase (MLCK), reducing myosin phosphorylation and consequently promoting vascular smooth muscle relaxation [26, 27]. The selective inhibitor of the sGC enzyme ODQ also inhibited the EAF-induced vasorelaxation. These results confirm the involvement of the NO/cGMP pathway in the EAF-induced endothelium-dependent relaxation.

In addition to NO production through the muscarinic receptor, endothelium cells can also induce relaxation by releasing prostaglandin I_2_ (PGI_2_, prostacyclin) through the cyclooxygenase pathway. PGI_2_ activates the prostaglandin I2 receptor, or just IP receptor, on the vascular smooth muscle cell membrane, coupled to adenylate cyclase through the protein (Gs), promoting an increase in cyclic adenosine monophosphate (cAMP) levels. cAMP levels have been a key cellular event to trigger blood vessel relaxation by IP agonists [25]. Increased concentrations of cGMP or cAMP are correlated with smooth muscle relaxation induced by various drugs and phytochemicals [28, 29]. However, neither the muscarinic receptor antagonism nor the cyclooxygenase inhibition was able to modify the EAF-induced vasodilation, suggesting that these pathways are not pivotal for the relaxant effect of this fraction.

The second pathway of PKG stimulation is the activation of the K^+^ channel [30]. The opening of a K^+^ channel in the vascular smooth muscle membrane cells causes hyperpolarization, increasing K^+^ efflux, leading to the closure of Ca^2+^ channels and, consequently, vasodilation [31]. TEA, at high concentrations, is a nonspecific K^+^ channel blocker that inhibits the ATP-, voltage-, and calcium-dependent subtypes of channels. Our results showed that the presence of TEA (10 mM) produced a significant reduction in the aortic relaxant response induced by the EAF, indicating the participation of K^+^ channels. Many subtypes of K^+^ channels have been identified in smooth muscle cells [32]. The contribution of each channel to vasodilation is estimated from the effect of selective inhibitors. Glibenclamide, which acts as a selective blocker of adenosine triphosphate- (ATP-) dependent K^+^ channels, and barium chloride, a selective blocker of inward-rectifier K^+^ channels, did not modify the EAF-induced relaxation, indicating that these K^+^ channel subtypes are not the main ones responsible for the fraction's actions. On the other hand, TEA (1 mM), which acts as a large-conductance Ca^2+^-activated K^+^ channel blocker, and 4-AP, a selective voltage-dependent K^+^ channel blocker, significantly reduced the vasorelaxation response of EAF. The voltage-dependent K^+^ channels are activated by depolarizing the membrane potential, resulting in repolarization and a return to the resting membrane potential, maintaining the resting vascular tone. The Ca^2+^-activated K^+^ channels are activated by either increased intracellular Ca^2+^ or membrane depolarization, resulting in the control of Ca^2+^ influx and K^+^ efflux [33]. Indeed, these subtypes of K^+^ channels seem to be the main ones for the fraction's vasodilating actions.

Our results suggest that EAF has bioactive compounds, which induce endothelium-dependent vasodilation, mainly through the NO pathway with consequent activation of K^+^ channels. The isolated rat aorta model has been a valuable tool to validate the vasoactive properties of extracts and compounds obtained from plants [34]. Extending these findings to the *in vivo* system, it was interesting to evaluate the effect of the EAF on the blood pressure values of rats. The data presented herein revealed that the treatment with EAF reduced the MAP, SAP, and DAP of both normotensive and hypertensive animals. In SHR, hypertension is generally attributed to the increased activity of the sympathetic nervous system, hyperactivation of the renin-angiotensin-aldosterone system, and endothelial dysfunction mainly due to the reduced bioavailability of NO [35, 36]. Thus, strategies aimed at reestablishing some of these pathways are essential tools for the therapeutic management of hypertension and associated conditions.

## 5. Conclusions

The present study shows that a semipurified fraction obtained from the *Leandra dasytricha* leaves has bioactive compounds that can synergistically induce vascular relaxation and, consequently, reduce blood pressure in normotensive and hypertensive animals. Further studies are required to confirm the mechanisms of action suggested in this study, which point to the involvement of the NO/cGMP pathway and K^+^ channel activation.

## Figures and Tables

**Table 1:** Chemical constituents from the ethyl acetate fraction (EAF) of *L. dasytricha.*

Peak	Compound	RT (min)	MF	UV (nm)	*m/z*		MS/MS
					[M + H]^+^	[M-H]^−^	
1	Di-O*-*hexoside	1.1	C_12_H_22_O_11_	—	365.1044^Na^	341.1028	—
2	Shikimic acid	1.2	C_7_H_10_O_5_	—	—	173.0459	—
3	Gallic acid^st^	2.4	C_7_H_6_O_5_	270	—	169.0125	—
4	Castalagin isomer	4.5	C_41_H_26_O_26_	285	935.0803	466.0275^–2^	466 ⟶ 301, 275, 231, 191, 169
5	Methyl gallate	7.8	C_8_H_8_O_5_	270	185.0444	183.0299	183 ⟶ 168
6	Catechin^st^	9.1	C_15_H_14_O_6_	270	291.0855	289.0695	289 ⟶ 221, 203, 151
7	Acutissimin A/B isomer	9.7	C_56_H_38_O_31_	285	—	602.0626^–2^	602 ⟶467, 301, 275, 249, 169
8	Tri-*O*-galloyl hexoside	15.6	C_27_H_24_O_18_	270	637.1035	635.089	635 ⟶ 465, 313, 423, 271, 211, 169
9	*O*-hexosyl phloretin	16.0	C_21_H_24_O_10_	284	437.1442	435.1297	435 ⟶ 255, 229
10	*C*-hexosyl hydroxyphlorizin	16.6	C_21_H_24_O_11_	284	453.1391	451.1246	451 ⟶ 361, 331, 239, 167
11	Ellagic acid^st^	17.7	C_14_H_6_O_8_	282, 368	303.0120	300.9996	301 ⟶ 283, 257, 245, 229
12	Di-*C*-hexosyl phloretin	19.1	C_27_H_34_O_15_	286	599.1970	597.1825	597 ⟶ 417, 387, 357, 315, 209, 167
13	*C*-hexosyl phloretin (nothofagin)	19.4	C_21_H_24_O_10_	286	437.1442	435.1297	435 ⟶ 345, 315, 273, 209, 167
14	Unknown	20.8	C_27_H_28_O_12_	280	545.1654	543.1508	543 ⟶ 313, 229, 169
15	*C*-hexosyl phloretin	24.2	C_21_H_24_O_10_	285	437.1437	435.1293	435 ⟶ 345, 315, 272, 209, 179, 167
16	Tri-*O*-methyl ellagic acid	24.4	C_17_H_12_O_8_	285, 368	345.0605	343.0459	345 ⟶ 330, 315, 285
17	*O*-galloyl *C*-hexosyl phloretin	24.8	C_28_H_28_O_14_	284	589.1536	587.1412	587 ⟶ 345, 315, 273, 209, 167
18	*O*-acetyl *C*-hexosyl phloretin	25.6	C_23_H_26_O_11_	284	479.1525	477.1407	477 ⟶ 345, 315, 273, 167
19	Phloretin	28.4	C_15_H_14_O_5_	285	275.0914	273.0768	273 ⟶ 189, 167, 151
20	Triterpene	33.8	C_30_H_48_O_5_	—	489.3575	487.3429	—
21	Triterpene	34.6	C_30_H_48_O_5_	—	489.3564	487.3445	—

MF: molecular formula; RT: retention time; ^Na^: [M+Na]^+^; ^−2^: [M-2H]^−2^; st: confirmed by authentic standard.

**Table 2:** Effects of EAF (0.3, 1, and 3 mg/kg) treatment on the blood pressure of normotensive rats (NTRs) and hypertensive rats (SHRs).

Groups	Vehicle	Acetylcholine 10 nmol/kg	EAF 0.3 mg/kg	EAF 1 mg/kg	EAF 3 mg/kg
NTR					
MAP (mm Hg)	—	−12.82 ± 3.07^*∗*^	−4.07 ± 1.99	−11.78 ± 3.55^*∗*#^	−24.20 ± 2.45^*∗*#^
SAP (mm Hg)	—	−12.44 ± 4.03^*∗*^	−3.47 ± 2.21	−12.20 ± 3.56^*∗*#^	−24.22 ± 2.30^*∗*#^
DAP (mm Hg)	—	−13.71 ± 2.03^*∗*^	−2.08 ± 1.49	−14.08 ± 3.94^*∗*#^	−25.36 ± 2.77^*∗*#^

SHR					
MAP (mm Hg)	—	-20.75 ± 6.86^*∗*^	−2.92 ± 1.68	−10.71 ± 2.32^*∗*#^	−21.17 ± 2.46^*∗*#^
SAP (mm Hg)	—	−20.92 ± 5.45^*∗*^	−4.09 ± 1.93	−10.70 ± 2.67^*∗*#^	−21.37 ± 3.29^*∗*#^
DAP (mm Hg)	—	−21.76 ± 6.69^*∗*^	−4.46 ± 1.33	−11.19 ± 1.75^*∗*#^	−21.35 ± 1.93^*∗*#^

The results show the mean ± S.E.M. of 6 animals in each group. Statistical analyses were performed by means of a one-way analysis of variance followed by Bonferroni's multiple comparison test. ^*∗*^*p* < 0.05 when compared to a vehicle, and ^#^*p* < 0.05 when compared to EAF 0.3 mg/kg or EAF 1 mg/kg. MAP, mean arterial pressure; SAP, systolic arterial pressure; and DAP, diastolic arterial pressure.

## Data Availability

Data are available from the corresponding author on request.

## Abbreviations

ACh: Acetylcholine
AHA: American Heart Association
4-AP: 4-Aminopyridine
BaCl_2_: Barium chloride
CVD: Cardiovascular disease
cGMP: Cyclic guanosine monophosphate
cAMP: Cyclic adenosine monophosphate
DAP: Diastolic arterial pressure
eNOS: Endothelium nitric oxide synthase
EDRF: Endothelium-derived relaxing factor
GLI: Glibenclamide
PKG: GMP-dependent protein kinase
ODQ: H-[1,2,3]oxadiazolo[4,3-alpha]quinoxalin
HR: Heart rate
MAP: Mean arterial pressure
MLCK: Myosin light-chain kinase
NO: Nitric oxide
L-NAME: N*ω*-nitro-l-arginine methyl ester
NTR: Normotensive rat
PE: Phenylephrine
PGI_2_: Prostaglandin I_2_
SAP: Systolic arterial pressure
sGC: Soluble guanylate cyclase
SHR: Spontaneously hypertensive rat
EAF: Ethyl acetate fraction
TEA: Tetraethylammonium.

## Supplementary Materials

Supplementary Materials Figure S1: molecular structure of nothofagin (**13**) identified in the ethyl acetate fraction from *L. dasytricha*.
