# Supplementary material for: Ethyl Acetate Fraction from Leandra dasytricha (A. Gray) Cong. Leaves Promotes Vasodilatation and Reduces Blood Pressure in Normotensive and Hypertensive Rats
Source: Evid Based Complement Alternat Med. 2021 Nov 15;2021:7203934. doi: 10.1155/2021/7203934 (PMC8608499; doi:10.1155/2021/7203934)
Supplement: Supplementary Materials — Figure S1: molecular structure of nothofagin (13) identified in the ethyl acetate fraction from L. dasytricha. [file 7203934.f1.docx]

**Supplementary material**

# Ethyl acetate fraction from *Leandra dasytricha* (A. Gray) Cong. leaves promotes vasodilatation and reduces blood pressure in normotensive and hypertensive rats

Rita de Cassia Vilhena da Silva^1^, Luísa Nathália Bolda Mariano^1^, Eleine Renata Bidinha^1^, Camila Leandra Bueno de Almeida^1^, Valdir Cechinel-Filho^1^, Vanessa Samudio Santos Zanuncio^2^, Denise Brentan Silva^2^, Arquimedes Gasparotto Junior^3^, Priscila de Souza^1*^

^1^Postgraduate Program in Pharmaceutical Sciences, Nucleus of Chemical-Pharmaceutical Investigations, University of Vale do Itajaí, Itajaí, Brazil.

^2^Laboratory of Natural Products and Mass Spectrometry, Faculty of Pharmaceutical Sciences, Food and Nutrition, Federal University of Mato Grosso do Sul, Campo Grande, MS, Brazil.

^3^Laboratory of Cardiovascular Pharmacology, Faculty of Health Sciences, Federal University of Grande Dourados, Dourados, MS, Brazil.

*Corresponding author:

Dr. Priscila de Souza

Postgraduate Program in Pharmaceutical Sciences, Nucleus of Chemical-Pharmaceutical Investigations.

University of Vale do Itajaí, 458 Uruguai St., 88302-202, Itajaí, SC, Brazil.

Phone: +55 (47) 3341-8160

E-mail: priscilasouza_rc@yahoo.com.br


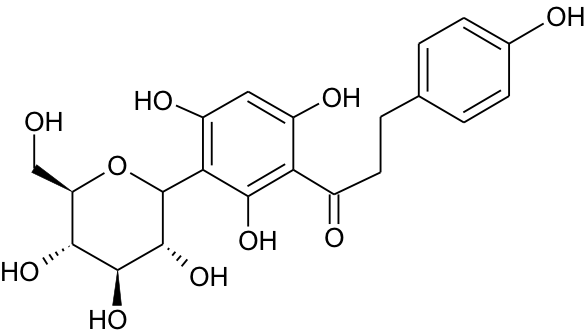


**Figure S1:** Molecular structure of nothofagin (**13**) identified in the ethyl acetate fraction from *L. dasytricha*.
